# A Computational Approach to Evaluate the Androgenic Affinity of Iprodione, Procymidone, Vinclozolin and Their Metabolites

**DOI:** 10.1371/journal.pone.0104822

**Published:** 2014-08-11

**Authors:** Corrado Lodovico Galli, Cristina Sensi, Amos Fumagalli, Chiara Parravicini, Marina Marinovich, Ivano Eberini

**Affiliations:** Dipartimento di Scienze Farmacologiche e Biomolecolari, Università degli Studi di Milano, Milano, Italia; University of Copenhagen, Denmark

## Abstract

Our research is aimed at devising and assessing a computational approach to evaluate the affinity of endocrine active substances (EASs) and their metabolites towards the ligand binding domain (LBD) of the androgen receptor (AR) in three distantly related species: human, rat, and zebrafish. We computed the affinity for all the selected molecules following a computational approach based on molecular modelling and docking. Three different classes of molecules with well-known endocrine activity (iprodione, procymidone, vinclozolin, and a selection of their metabolites) were evaluated. Our approach was demonstrated useful as the first step of chemical safety evaluation since ligand-target interaction is a necessary condition for exerting any biological effect. Moreover, a different sensitivity concerning AR LBD was computed for the tested species (rat being the least sensitive of the three). This evidence suggests that, in order not to over−/under-estimate the risks connected with the use of a chemical entity, further *in vitro* and/or *in vivo* tests should be carried out only after an accurate evaluation of the most suitable cellular system or animal species. The introduction of *in silico* approaches to evaluate hazard can accelerate discovery and innovation with a lower economic effort than with a fully wet strategy.

## Introduction

During the last years, following some evidence suggesting that exposure to environmental chemicals can lead to disruption of endocrine function in a number of wildlife species (molluscs, crustacean, fish, and birds), concern has been expressed also for human health. Even if many EU regulations contain specific provisions on chemicals that can affect the endocrine system, (e.g. REACH [1], Plant Protection Products Regulation [2], Biocides Regulation [3], Regulation on cosmetics [4], Water Framework Directive [5]), warning was raised by Bars et al. [6], who stated that recent European legislation has created a hazard-based approval criterion, which supports marketing and use of chemicals only on the basis that they do not induce endocrine activation in humans or wildlife species.

The *in silico* approaches have become relevant to these legislations as far as they can help reduce the number of animals used (by pre-screening and prioritising chemicals for more intensive testing). Moreover, they are in line with the vision of the 21^st^ century toxicity paradigm: chemicals will be subjected to a multiplicity of high-throughput screening tests to detect cellular response to an array of “pathways of toxicity”, and results will feed into computational systems biology tools that model dose-response effects and inform new risk assessment approaches [7]. Several computational approaches may be useful for evaluating interactions between a receptor and its putative ligands: some of them are based on molecular docking, which has been reliably used for decades in pharmacological research and development [8], [9].

The present research is aimed at devising and assessing a computational approach to evaluate the affinity towards the androgen receptor (AR) of hormonally active substances and of their metabolites. To build the model, the ligand binding domain (LBD) structures of ARs of three distantly related species (human, rat, and zebrafish) were used. The use of three reference species was also meant to evaluate whether their sensitivities to the test chemicals do differ. Three fungicides (vinclozolin, iprodione and procymidone) and their rat metabolites [10]–[13], all with a well-established androgenic activity [14]–[18], were tested. The proposed model can anticipate, very early in the hazard identification procedure, the ability of a chemical to bind the AR LBD. This information is very useful to set up a priority list during the screening of a large chemical database, and may be exploited also to design new chemicals for use in different fields [19]. To date, considering the available tests, evidence coming just from *in silico* assays cannot be considered sufficient for the identification of an endocrine-active substance, and the assessment of its possible relevance to humans. However, the information about qualitative and quantitative hormonal response to a chemical might be useful to identify its ‘potential’ for interaction with the endocrine system, and therefore to better design and carry out further testing steps.

## Materials and Methods

### Comparative modeling

The human and rat AR LBD crystal structures were downloaded from the RCSB Protein Data Bank [PDB entry: 3L3X (chain A), and PDB entry: 1I37, respectively]. The crystallographic structures of the human and rat receptors were then submitted to a preparation step, based on energy minimization (EM) with the Amber12:EHT force field [20] and the reaction field solvation model. Refinement was carried out down to a Root Mean Square (RMS) gradient of 0.05 kcal/mol/Å^2^. All the computational procedures were carried out with the Molecular Operating Environment (MOE).

The zebrafish AR sequence was downloaded from the UniProt Protein Knowledgbase database [entry: B9P3Q7]. 1T7R, corresponding to the chimpanzee AR LBD, was set as template in order to compute a 3D structural model [21]. The alignment produced by the MOE Align program with default parameters was manually adjusted. Comparative model building was carried out with the MOE Homology Model program. Ten independent models were built and refined, then the highest scoring intermediate model - according to the electrostatic solvation energy calculated using a Generalized Born/Volume Integral (GB/VI) methodology [22] - was submitted to a further round of EM. Both for the intermediate and the final structures, the refinement procedures consisted in EM runs based on the Amber12:EHT force field with the reaction field solvation model. The quality of the final model was carefully checked with the MOE Protein Geometry module, in order to make sure that the Ramachandran plot, the side chain packing, and the stereochemical quality of the generated structure were acceptable.

### Binding site analysis

The binding site of each receptor was identified through the MOE Site Finder program, which uses a geometric approach to calculate putative binding sites in a protein, starting from its tridimensional structure. This method is not based on energy models, but only on alpha spheres, which are a generalization of convex hulls [23]. The prediction of the binding sites, performed by the MOE Site Finder module, confirmed the binding sites defined by the co-crystallized ligands in the *holo-*forms of the investigated proteins.

### Molecular database preparation

**Figure 1:**
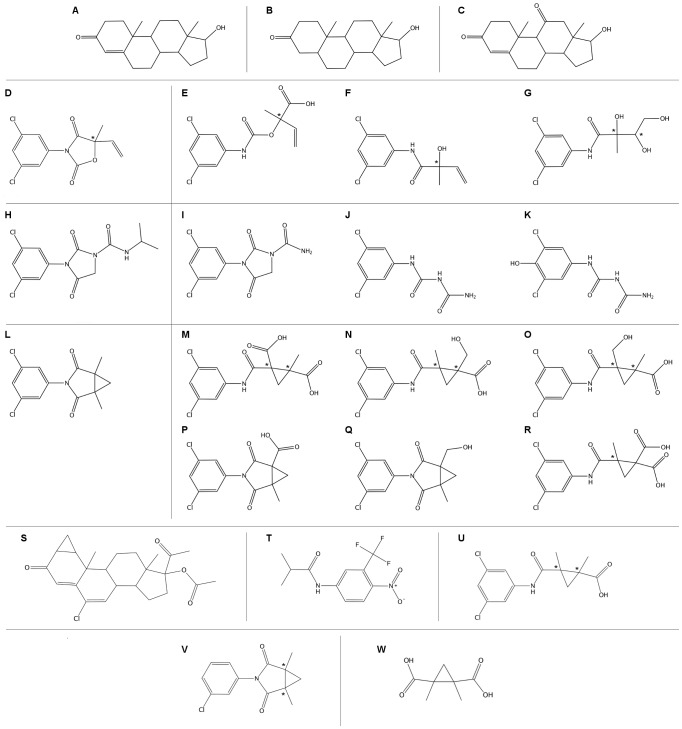
2D structures of the molecules used to build the test database. The *stereogenic* centres are marked with an asterisk (*). Each molecule is marked by a letter: A = testosterone, B = dihydrotestosterone (DHT), C = 11-ketotestosterone, D = vinclozolin, E = vinclozolin_1, E = vinclozolin_2, F = vinclozolin_5, H = iprodione, I = iprodione_8, J = iprodione_13, K = iprodione_14, L = procymidone, M = procymidone_1, N = procymidone_2, O = procymidone_3, P = procymidone_4, Q = procymidone_5, R = procymidone_6, S = cyproterone acetate, T = flutamide, U = procymidone-NH-COOH, V = procymidone-3-Cl, W = cyclopropane-(COOH)_2_.

The database was prepared by building with the MOE Builder the molecular structures of the three fungicides and of their major rat metabolites, as well as the molecular structures of the endogenous hormones in each species (human, rat and zebrafish). Each structure was converted into a tridimensional structure, and energy was minimized, with the MOE Energy Minimize program and the Amber12:EHT force field, down to a RMS gradient of 0.05 kcal/mol/Å^2^. Since some of these molecules contain *stereogenic* centres (see Figure 1, atoms marked by an asterisk), all the possible enantiomers/diastereomers were built and added to the database. Moreover, 20,000 conformations were generated for each ligand by sampling all their rotatable bonds.

### Molecular docking

The *in silico* screening was carried out with the MOE Dock program, part of the MOE Simulation module. The whole procedure was carried out for each of the three AR LBD - human, rat and zebrafish. The AR LBD was set as ‘Receptor’. The selected placement methodology was ‘Triangle Matcher’, which is the best method for standard and well-defined binding sites. With Triangle Matcher the poses are generated by superposing triplets of ligand atoms and triplets of receptor site points. The receptor site points are alpha spheres centres that represent locations of tight packing. Thirty complexes were generated for each tested ligand. Duplicate complexes were then removed: poses are considered as duplicates if the same set of ligand-receptor atom pairs are involved in hydrogen bond interactions and the same set of ligand atom receptor residue pairs are involved in hydrophobic interactions. The accepted poses were scored according to the London dG scoring function, which estimates the binding free energy of the ligand from a given pose.
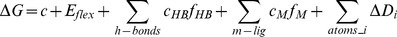
(1)where *c* represents the average gain/loss of rotational and translational entropy; *E_flex_* is the energy due to the loss of flexibility of the ligand (calculated from ligand topology only); *f_HB_* measures geometric imperfections of hydrogen bonds and takes a value in [0,1]; *c_HB_* is the energy of an ideal hydrogen bond; *f_M_* measures geometric imperfections of metal ligations and takes a value in [0,1]; *c_M_* is the energy of an ideal metal ligation; and *D_i_* is the desolvation energy of atom *i*. The difference in desolvation energies is calculated according to the formula
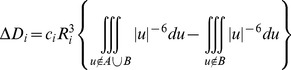
(2)where *A* and *B* are the protein and/or ligand volumes with atom *i* belonging to volume *B*; *R_i_* is the solvation radius of atom *i* (taken as the OPLS-AA van der Waals sigma parameter plus 0.5 Å); and *c_i_* is the desolvation coefficient of atom *i*. The coefficients (*c*, *c_HB_*, *c_M_*, *c_i_*) have been fitted from approx. 400 x-ray crystal structures of protein–ligand complexes with available experimental p*K_i_* data. Atoms are categorized into about a dozen types for the assignment of the *c_i_* coefficients. The triple integrals are approximated using Generalized Born integral formulas.

All the saved solutions were submitted to a further refinement step, based on molecular mechanics (MM). In order to speed up the calculation, residues over 6 Å cutoff distance away from the pre-refined pose were ignored, both during the refinement and in the final energy evaluation. All receptor atoms were held fixed during the refinement. During the course of the refinement, solvation effects were calculated using the reaction field functional form for the electrostatic energy term. The final energy, docking score, was evaluated using the GBVI/WSA dG scoring function with the Generalized Born solvation model (GBVI) [24]. The GBVI/WSA dG is a forcefield-based scoring function, which estimates the free energy of binding of the ligand from a given pose. It has been trained using the MMFF94x and AMBER99 forcefields on the 99 protein-ligand complexes of the Solvated Interaction Energy (SIE) training set [25]. The functional form is a sum of terms:
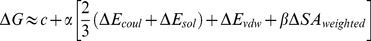
(3)where *c* represents the average gain/loss of rotational and translational entropy. *α* and *β* are constants, which were determined during training (along with *c*) and are forcefield-dependent. *E_coul_* is the coulombic electrostatic term, which is calculated using currently loaded charges, using a constant dielectric of 1. *E_sol_* is the solvation electrostatic term, which is calculated using the GB/VI solvation model. *E_vdw_* is the van der Waals contribution to binding. *SA_weighted_* is the surface area weighted by exposure. This weighting scheme penalizes exposed surface area. All the ligands of the molecular database were tested according to the above procedure. The Amber12:EHT force field was used for all the computational procedures.

Docking accuracy was evaluated using the present procedure for reproducing 10 ligand-receptor crystallographic complexes. Ligand RMSD values between crystallographic *vs* computational complexes were measured.

As a negative dataset, we randomly selected 1,000 compounds from the Asinex Platinum Database (http://www.asinex.com) and docked them on the human AR LBD, using the above procedure.

### Low-mode molecular dynamics simulations

For studying the flexibility of AR LBD helix 12, we applied the low-mode molecular dynamics approach, aimed at focusing a MD trajectory along the low-mode vibrations and featuring a very efficient way *vs* classical MD for searching for minima troughs on the potential energy surface. To run these computations, we used the MOE Conformational Search program of the Conformations module. This program uses an efficient implicit method for estimating the low-frequency modes and is based on the attenuation of high-range velocities as described in detail in [26].

The human AR LBD bound to: i) dihydrotestosterone (DHT), an agonist, ii) cyproterone acetate, an antagonist, and iii) in its *apo* form was simulated after preparation. The complex with DHT was obtained from RCSB PDB (3L3X); the *apo* form was obtained by *in silico* removing DHT, and the complex with cyproterone acetate was obtained through molecular docking on the same crystal structure.

Both helix 12 (set as a rigid body) and the loop joining helix 12 to the preceding helix were left free to move during the low-mode molecular dynamics, whereas the residues more than 4.5 Å away were fixed (not free to move, but used for the energy calculations); the other residues were defined as inert (fixed and not used for energy calculations). The simulation was carried out with default parameters, except for strain energy cutoff, which was set at 100 kcal/mol. One thousand conformations were generated and analysed.

The same computational approach was used also to produce ensembles of natural ligand-receptor complexes, in order to estimate with greater accuracy their binding free energies. To this purpose, we started from the top scoring poses obtained from the molecular docking procedure. The ligand and the residues within 4.5 Å from it were left free to move during the low-mode molecular dynamics, whereas the residues more than 4.5 Å away were fixed (see above); the other residues were defined as inert (see above). The simulation was carried out with default parameters, except for strain energy cutoff, which was set at 50 kcal/mol. Four hundred conformations were generated and the one with the lowest energy was used to compute the complex dissociation constant value. The Amber12:EHT force field was used for all the computational procedures.

### Dissociation constant calculation

The estimated binding affinity of the top-scoring solution for each complex (receptor-ligand) was not directly computed from the GBVI/WSA dG value, but the complexes were further refined through the use of a set of specific MOE procedures, named LigX, aimed at the minimization of ligands in the receptor binding site. The dissociation constant (*K_i_*) was computed through the binding free energy estimated with the GBVI/WSA dG scoring function, after complex optimization with LigX, according to the following equation:
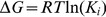
(4)where *R* represents the gas constant and *T* the temperature. The *K_i_* was computed starting from the binding free energy values at a fixed temperature (300 K).

## Results

### Comparative modeling

**Figure 2:**
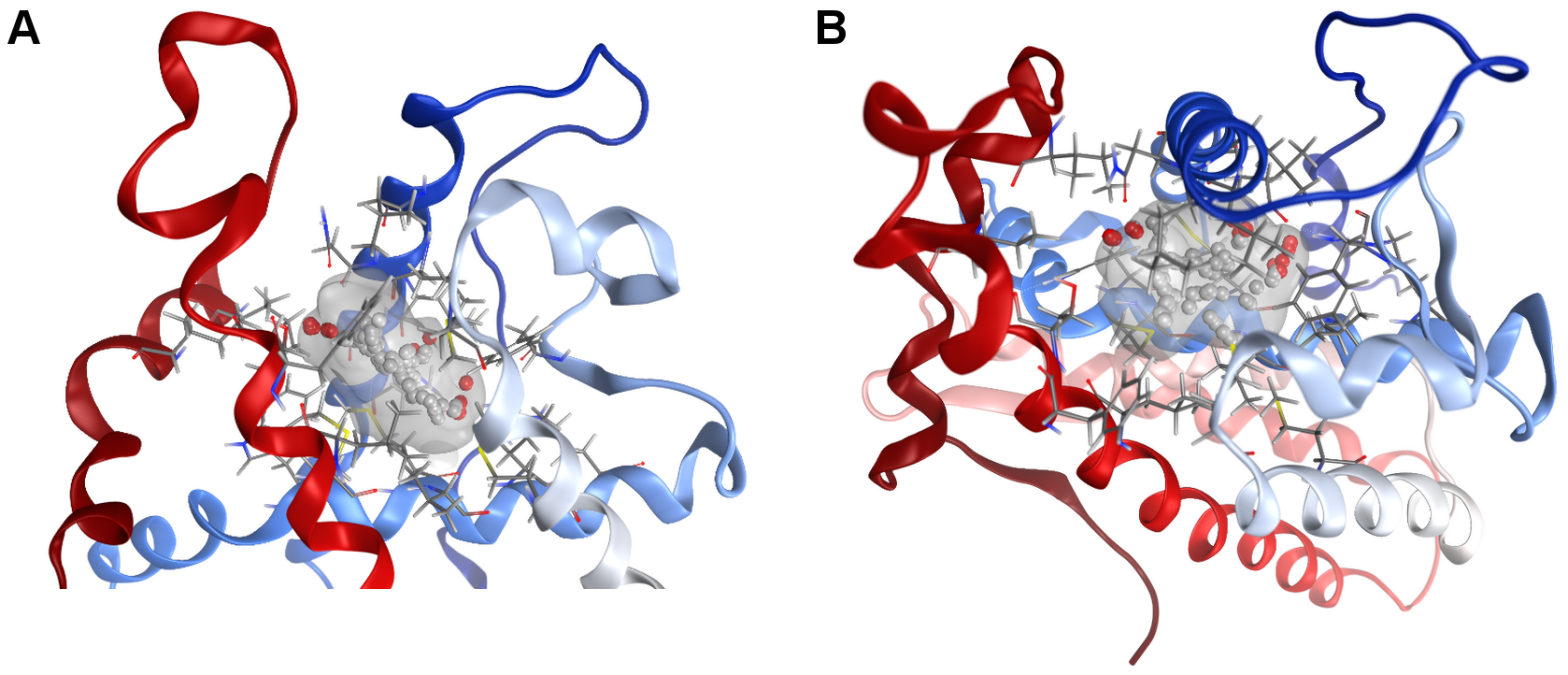
Molecular surface of the binding site and filling dummy atoms in the zebrafish AR LBD model, side (A) and top (B) view.

**Figure 3:**
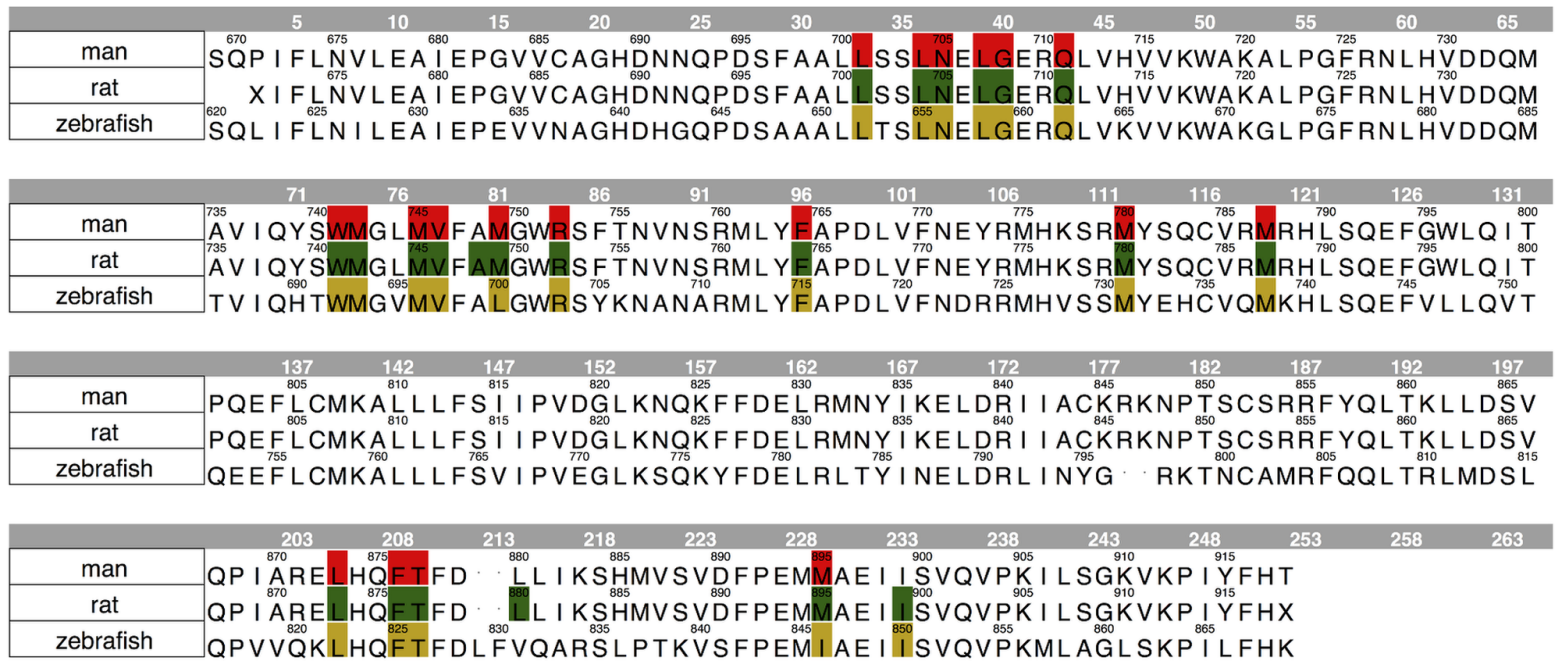
Global alignment of the selected AR LBD: the residues of the binding sites are highlighted with the following color-code: red for human AR, green for rat AR, and yellow for zebrafish AR.

The homology model of the zebrafish AR LBD was built using as template 1T7R, the crystal structure of chimpanzee AR LBD (66% sequence identity). Figure S1 in File S1 shows the alignment used for carrying out the modelling procedure. Ten independent models were built and refined, and the one top scoring according to the electrostatic solvation energy was selected. The presence of a well-defined binding site, shown in Figure 2, was probed through the MOE Site Finder program. The same approach was applied to human and rat AR LBD crystals. Table 1 reports the binding site scores for the three receptor structures and lists the residues lining each of them. Figure 3 shows the global alignment of the investigated AR LBD; the residues in the binding sites are highlighted. Finally, after a structural superposition, the global and the binding site RMSD values were computed both for α-carbons and for whole residues of the three AR LBD; data are summarized in Table 2.

**Table 1:** Binding site features for each of the three selected receptors.

AR	Size	PLB	Hyd	Side	Residues
Human	54	2.43	24	44	LEU701 LEU704 ASN705 LEU707 GLY708 GLN711 TRP741 MET742 MET745 VAL746 MET749 ARG752 PHE764 MET780 MET787 LEU873 PHE876 THR877 MET895
Rat	70	3.01	35	54	LEU701 LEU704 ASN705 LEU707 GLY708 GLN711 TRP741 MET742 MET745 VAL746 ALA748 MET749 ARG752 PHE764 MET780 MET787 LEU873 PHE876 THR877 LEU880 MET895 ILE899
Zebrafish	73	2.44	30	47	LEU652 LEU655 ASN656 LEU658 GLY659 GLN662 TRP692 MET693 MET696 VAL697 LEU700 ARG703 PHE715 MET731 MET738 LEU822 PHE825 THR826 ILE846 ILE850

*Size* indicates the number of alpha spheres comprising the site. *PLB* is the Propensity for Ligand Binding score for the contact residues. *Hyd* indicates the number of hydrophobic contact atoms in the receptor. *Side* indicates the number of sidechain contact atoms in the receptor.

**Table 2:** AR LBD and binding site RMSD values, computed for α-carbons and for whole residues of the three selected receptors.

	RMSD	RMSD
	C alpha [Å]	All atoms [Å]
AR LBD	1.27	1.52
Binding site	0.59	0.77

### Validation of the docking protocol

The accuracy of the docking protocol detailed under Methods was extensively validated by reproducing the ligand-receptor complexes for 10 different AR LBDs deposited in the RCSB PDB. Table 3 reports the selected structures and the RMSD values between the co-crystallized and the docked ligands: the latter range between 0.13 and 0.35 Å.

**Table 3:** Molecular docking validation dataset and RMSD values between the co-crystallized and the docked ligands.

Species	PDB code	Ligand	RMSD [Å]
Human	3L3X	DHT	0.17
Human	2AMA	DHT	0.24
Human	2PIO	DHT	0.26
Human	2AM9	testosterone	0.13
Human	2YHD	testosterone	0.26
Human	2OZ7	cyproterone acetate	0.35
Chimpanzee	1T7R	DHT	0.15
Chimpanzee	1T73	DHT	0.14
Rat	1I37	DHT	0.32
Rat	3G0W	oxazolidin-2-imine	0.30

In order to assess the correlation between docking scores and biological data, we docked to the rat AR LBD seven ligands from a published dataset, whose relative affinities for the rat AR binding site had been experimentally determined [27]. The computed relative affinities (dissociation constants, K_i_) showed the same ranking as the experimental ones; Table 4 reports experimental (literature data) *vs in silico* (our computations) data.

**Table 4:** Experimental (from [27]) and *in silico* (computed) dissociation constants for the selected compounds with respect to rat AR binding site.

Letter (Fig. 1)	Natural ligand	Relative binding affinity	ΔG-MD kcal/mol	Computed K_i_
B	DHT	100	−12.52	1.6·**10^−9^**
S	cyproterone acetate	14	−12.47	1.5·**10^−9^**
T	flutamide	0.058	−8.78	2.89·**10^−6^**
L	procymidone	0.065	−8.66	2.36·**10^−6^**
V	(R,S)-procymidone-3-Cl	0.050	−8.38	1.47·**10^−6^**
U	(R,S)-procymidone-NH-COOH	<0.0001	−8.37	1.44·**10^−6^**
W	cyclopropane-(COOH)_2_	<0.0001	−6.12	3.19·**10^−4^**

ΔG-MD: binding free energy computed through molecular docking.

Furthermore, from an Asinex combinatorial chemistry dataset, we randomly selected 1,000 compounds, which have never been predicted/demonstrated to bind AR LBDs, and evaluated their docking score on the human AR. All of their docking scores were positive (>0, as plotted in Figure S2 in File S1), which substantiates the ability of the proposed procedure to correctly identify negative (non-interacting) compounds.

### Results of docking the test compounds to zebrafish, rat and human AR LBD

Figure 1 reports the structures of the three parent test fungicides and of their selected metabolites. The molecular database also contains the main endogenous androgenic hormones for all the three species, namely testosterone and DHT for human and rat [28], testosterone and 11-ketotestosterone for zebrafish [29].

**Figure 4:**
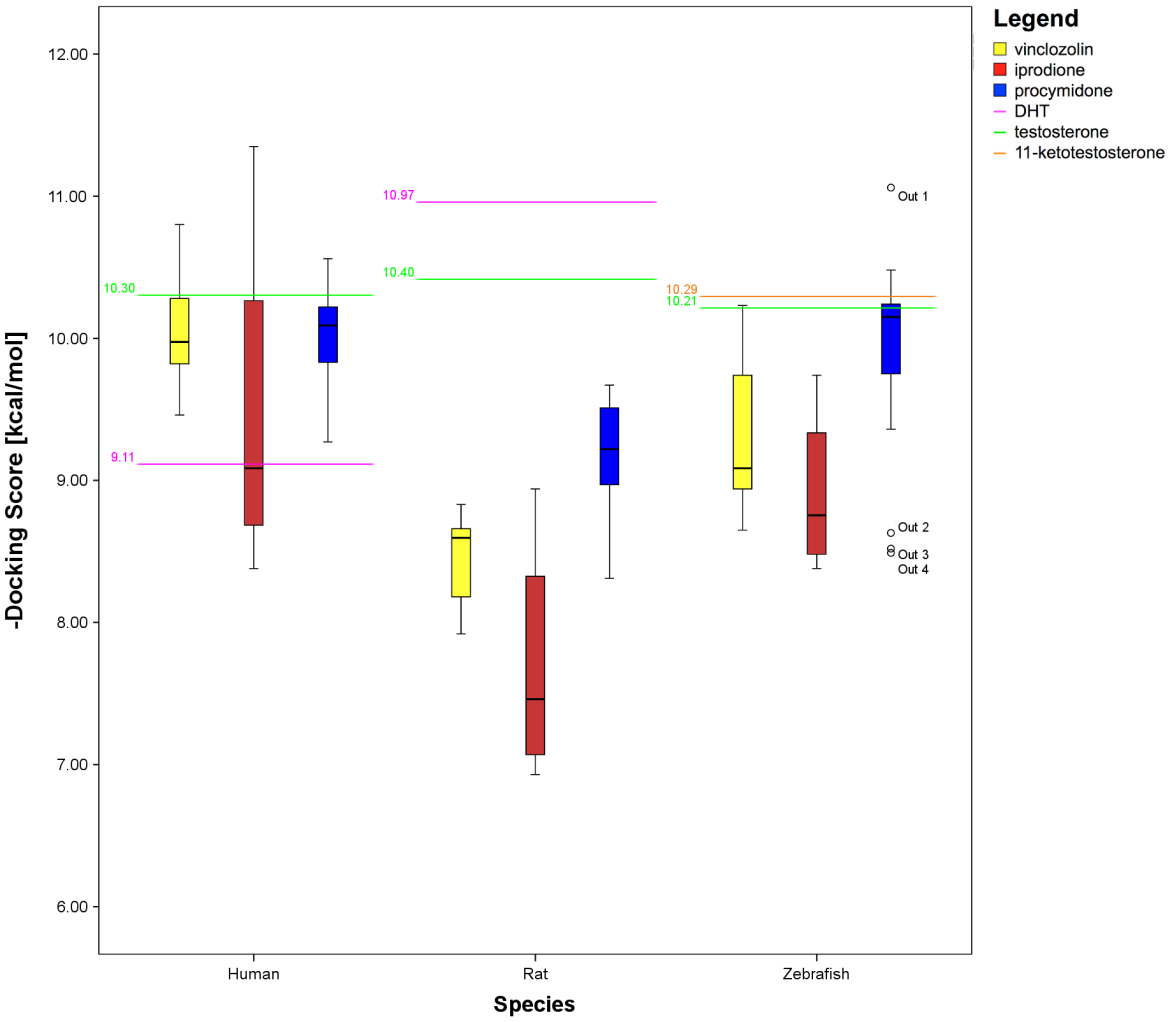
Box plot for all the calculated binding free energies. The yellow boxes represent the binding free energies of the vinclozolin and its metabolites for each species. The red boxes represent the binding free energies of iprodione and its metabolites for each species. The blue boxes represent the binding free energies of procymidone and its metabolites for each species. Outliers are marked as circles. Binding free energies of the endogenous hormones are marked with continuous lines.

Table 5 reports the dissociation constants (K_i_) computed complexes through equation (4) (see under Methods) for all AR LBD. From these data, endogenous hormones show the highest affinity for their AR in rat; instead, both in zebrafish and humans, the affinity of endogenous hormones for their AR is lower than with some xenobiotics. Table 6 reports the Docking Score (kcal/mol) for the tested chemicals. For human AR, in comparison with the hormones, (S)-vinclozolin and (R)-vinclozolin show an intermediate affinity (−9.87 kcal/mol and −9.61 kcal/mol, respectively), whereas their metabolites, (R)-vinclozolin_1 and (S)-vinclozolin_1, show the second (−10.80 kcal/mol) and the fourth highest affinity (−10.44 kcal/mol), respectively. Iprodione shows the highest affinity (−11.35 kcal/mol) for human AR, whereas procymidone one of the lowest (−9.27 kcal/mol). The Docking Scores reported in Table 6 were used to build the box plot reported in Figure 4.

**Table 5:** Experimental (from literature) and *in silico* (computed) dissociation constants for the three endogenous tested hormones.

Species	Natural ligand	DS kcal/mol	ΔG-LM kcal/mol	ΔG-MD kcal/mol	K_i_ (from MD)	Experimental *K_i_*
human	testosterone [45]	−10.30	−12.08	−12.49	1.55·**10^−9^**	Low nM range
human	DHT [46]	−9.11	−11.10	−12.08	7.76·**10^−8^**	1.3·**10^−9^**±0.2·**10^−9^**
human	DHT [47]	−9.11	−11.10	−12.08	7.76·**10^−8^**	2.3·**10^−10^**±0.4·**10^−10^**
zebrafish	testosterone [28]	−10.21	−12.06	−12.19	9.35·**10^−8^**	1.70·**10^−9^**±0.50·**10^−9^**
zebrafish	11-ketotestosterone [28]	−10.29	−12.09	−10.93	1.11·**10^−8^**	4.77·**10^−9^**±2.26·**10^−9^**
rat	testosterone	−10.40	−12.25	−12.81	2.67·**10^−9^**	NA
rat	DHT [48]	−10.97	−12.37	−12.52	1.64·**10^−9^**	2.7·**10^−10^**

DS: Docking Score; ΔG-LM: binding free energy computed through low-mode molecular dynamics simulations; ΔG-MD: binding free energy computed through molecular docking; K_i_: dissociation constant computed from molecular docking data.

**Table 6:** Docking Score (DS) (kcal/mol) for the top scoring poses (protein-ligand complexes) for all the compounds of the tested database.

		Human DS	Rat DS	Zebrafish DS
Letter (Fig. 1)	Molecule	kcal/mol	kcal/mol	kcal/mol
D	(R)-vinclozolin	−9.61	−8.22	−9.12
D	(S)-vinclozolin	−9.87	−8.18	−8.73
E	(R)-vinclozolin_1	−10.80	−8.82	−9.86
E	(S)-vinclozolin_1	−10.44	−8.83	−10.23
F	(R)-vinclozolin_2	−9.46	−7.96	−8.94
F	(S)-vinclozolin_2	−9.82	−7.92	−8.65
G	(R,R)-vinclozolin_5	−10.04	−8.62	−9.04
G	(R,S)-vinclozolin_5	−10.01	−8.66	−9.74
G	(S,R)-vinclozolin_5	−10.28	−8.57	−9.26
G	(S,S)-vinclozolin_5	−9.94	−8.62	−9.05
H	iprodione	−11.35	−8.94	−9.74
I	iprodione_8	−9.18	−7.71	−8.93
J	iprodione_13	−8.38	−6.93	−8.38
K	iprodione_14	−8.99	−7.21	−8.58
L	procymidone	−9.27	−8.31	−8.49
M	(R,R)-procymidone_1	−9.86	−9.51	−9.94
M	(R,S)-procymidone_1	−10.56	−9.44	−10.24
M	(S,R)-procymidone_1	−10.20	−9.35	−10.35
M	(S,S)-procymidone_1	−10.31	−9.60	−9.75
N	(R,R)-procymidone_2	−9.83	−8.87	−10.47
N	(R,S)- procymidone_2	−10.17	−9.17	−9.85
N	(S,R)-procymidone_2	−10.09	−8.97	−10.17
N	(S,S)-procymidone_2	−10.21	−9.19	−10.24
O	(R,R)-procymidone_3	−9.96	−9.67	−11.06
O	(R,S)-procymidone_3	−9.91	−9.50	−9.36
O	(S,R)- procymidone_3	−9.53	−9.15	−9.84
O	(S,S)-procymidone_3	−10.22	−9.22	−10.15
P	procymidone_4	−9.74	−8.49	−8.52
Q	procymidone_5	−9.54	−8.37	−8.63
R	(R)-procymidone_6	−10.29	−9.65	−10.23
R	(S)-procymidone_6	−10.28	−9.58	−10.48
A	testosterone	−10.30	−10.40	−10.21
B	DHT	−9.11	−10.97	–
C	11-ketotestosterone	–	–	−10.29

The last three rows contain the DS values for the endogenous hormones in each species.

Taking as thresholds the affinities for the endogenous hormones [30], [31], five molecules, iprodione, (R)-vinclozolin_1, (R,S)-procymidone_1, (S)-vinclozolin_1 and (S,S)-procymidone_1, show a higher affinity for human AR than testosterone (−10.30 kcal/mol) (Figure 4, human, green line), while only two molecules, iprodione_14 and iprodione_13, have a lower affinity (Figure 4, human, pink line) than DHT (−9.11 kcal/mol).

In rat, iprodione and its metabolites have the lowest affinity (higher Docking Score) for AR: iprodione_13 (J), −6.93 kcal/mol, and iprodione_14 (K) −7.21 kcal/mol, rank worse than the parent molecule iprodione (−8.94 kcal/mol). The affinity varies extensively among molecules derived from procymidone: (R)-procymidone_6 (R), −9.65 kcal/mol, and (R,R)-procymidone_3 (O), −9.67 kcal/mol, have a favourable binding energy, contrary to the parent compound, procymidone (L), −8.31 kcal/mol. Compared with their metabolites, (S)-vinclozolin and (R)-vinclozolin show low affinities for rat AR: −8.22 kcal/mol and −8.18 kcal/mol, respectively. Finally, the binding energies of the endogenous hormones are the lowest (most favourable) among the tested molecules: testosterone, −10.40 kcal/mol, and DHT, −10.97 kcal/mol.

In zebrafish, procymidone metabolites vary extensively in energy: (R,R)-procymidone_3 (O) has the best affinity (−11.06 kcal/mol), but procymidone_4 (P) has the third worst energy (−8.52 kcal/mol), and the parent compound has a low affinity (−8.49 kcal/mol) for AR.

As in rat, (R)-vinclozolin, −9.12 kcal/mol, and (S)-vinclozolin, −8.73 kcal/mol, are in the low ranking positions. Also iprodione, −9.74 kcal/mol, and its metabolites show low affinity for zebrafish AR: the molecule with the lowest affinity is iprodione_13 (J), −8.38 kcal/mol.

Using hormone affinity as threshold, four molecules, all procymidone metabolites, (R,R)-procymidone_3, (S)-procymidone_6, (R,R)-procymidone_2, (S,R)-procymidone_1, display a better affinity than 11-ketotestosterone (−10.29 kcal/mol), while testosterone shows a slightly lower affinity (−10.21 kcal/mol).

**Figure 5:**
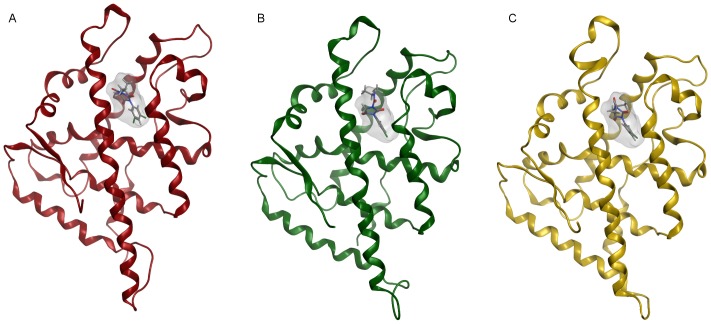
Best pose of iprodione complexed with the AR LBD in each species. The ligand molecular surface is also rendered. A) Iprodione complexed with the human AR LBD, B) iprodione complexed with the rat AR LBD, and C) iprodione complexed with zebrafish AR LBD.

Only for exemplification purposes, Figure 5 reports the top scoring poses for iprodione complexed with human (A), rat (B), and zebrafish (C) AR, respectively; iprodione orientation appears similar in the three complexes, with a RMSD value of 0.8 Å between human and zebrafish, and 1.9 Å between rat and zebrafish.

### Low-mode molecular dynamics simulations

Differences between experimental and computational values of *K_i_* are consistently expected for nuclear steroid receptors and are specifically connected with the dynamic changes in their structure. After the initial molecular recognition step, nuclear steroid receptors deeply rearrange [32]–[34], partially blocking the ligand into their binding site through a displacement of helix 12; as a result, apparent *K_i_* measured through experimental approaches are very low. Our *in silico* approach does not take into account the receptor rearrangement and this results in higher values for computed K_i_s.

Low-mode molecular dynamics simulations of AR LBDs were then run, under different computational setups, with a twofold aim: i) sampling the conformational space of helix 12 in the human AR LBD, when bound to an agonist and to an antagonist, and in its *apo* form); ii) estimating binding affinities of the natural hormones for the three AR LBDs at a higher accuracy level than with molecular docking.

**Figure 6:**
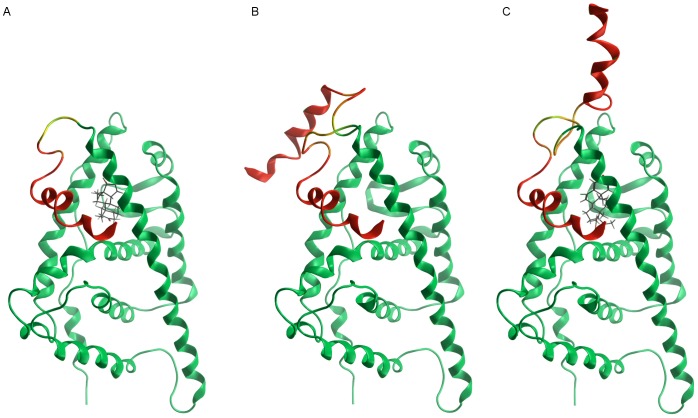
Low-mode molecular dynamics simulations. Superposition of the starting conformation (helix 12) and the most energetically favoured open conformations for the agonist-bound LBD (A), the *apo* LBD (B), and the antagonist-bound LBD (C).

During the molecular dynamics simulations, helix 12 keeps a closed conformation when the human AR binds an agonist (DHT), whereas it opens when the LBD is empty or bound to an antagonist (cyproterone acetate). Starting from a common closed conformation of helix 12 in all the three setups, only the *apo* and the antagonist-bound structures rapidly evolve towards helix 12 opening. Figure 6 shows the three closed starting conformations (helix 12), superposed to the most energetically favoured open conformation for the *apo* (Figure 6, panel B) and the antagonist-bound LBD (Figure 6, panel C). On the contrary, helix 12 does not open (1,000 generated and analysed conformations) when LBD is bound to an AR agonist, such as DHT (Figure 6, panel A). In spite of the ability of molecular dynamics to correctly sample the reported helix 12 conformational transition, the differences between experimental and computational dissociation constant values for natural agonists cannot yet be compensated for. Indeed, as shown in Table 5, the binding affinities of the natural hormones for the three investigated LBDs, computed applying LigX to the lowest energy complex out of 400 obtained from the low-mode molecular dynamics simulations, are very close to the affinities obtained from our rapid docking procedure. The experimental issue is connected with the definition of *K_i_* as the ratio k_off_/k_on_, where k_off_ is the dissociation rate constant in min^−1^ and k_on_ is the association rate constant in M^−1^ min^−1^. The closed conformation of helix 12 induced by the binding of an agonist produces a decrease in k_off_ values, thus reducing the apparent dissociation constant. The discrepancy between experimental and computational *K_i_* for the tested natural agonists is strictly connected with this phenomenon. The analysis of the interactions between helix 12 and DHT, carried out on a crystallographic complex (RCSB PDB code: 3L3X), shows only one weak and non-specific interaction between the ligand and the side chain of Met 895, in helix 12 (see Figure S3 in File S1), corroborating a kinetic more than a thermodynamic effect as the reason for the discrepancy between computed and experimental K_i_s.

## Discussion

Traditional *in vitro* and *in vivo* toxicity-testing strategies, which are expensive and time consuming and involve a large number of test animals, have been evolving over the last few decades, in order to address increasing concerns about a wider variety of toxic responses, such as subtle neurotoxic effects, adverse immunologic changes, and endocrine activity. Moreover, toxicity testing is under increasing pressure, and the most utilized approach, which relies primarily on *in vivo* mammalian toxicity testing, is unable to adequately meet the competing demands [35].

Although the current knowledge may not yet allow to fully eliminating the need for *in vivo* testing, our proposed computational approach, associated with suitable *in vitro* assays, can provide an effective tool to identify, at a very early stage, the potency of any EAS, through the measurement of its affinity (the binding free energy/complex K_i_) for the AR. This kind of information can be useful at the very beginning of the pipeline of hazard identification for compounds with putative EAS activity. The direct interaction between the putative EAS and the AR is a prerequisite to biological activity and should be carefully kept into account, as done in our model. Interaction depends principally upon affinity, which is a term referring to the strength of interaction between two molecules. The affinity of a ligand for a specific receptor determines its residence time of association, a parameter often quantified by the dissociation constant [36]. Generally, the higher is the affinity the longer is the residence time. Low affinity ligands do not need any evaluation of intrinsic activity (α, which is the relative ability of a drug-receptor complex to produce a maximum functional response), since they do not spend enough time in the receptor binding site to exert any effect either in *in vitro* or in *in vivo* tests.

Although interaction between ligand and receptor is essential in order to cause any effect, the intrinsic activity is the key to the ability of a molecule to induce a specific response. For the molecules with *in silico* high affinity for ARs, *in vitro* selected tests should first confirm the computed affinity. Next, only for the molecules that show high *in vitro* affinity, the intrinsic activity should be evaluated in order to discriminate between agonist (α = 1), partial agonist (1< α <0), or antagonist (α = 0).

Our *in silico* approach can thus compute the affinity of the simulated complexes, whereas reliable values for intrinsic activity (α) can be obtained only by *in vitro* and/or *in vivo* tests [37], [38].

Nowadays, several *in vitro* tests aim at evaluating the affinity of chemicals for ARs, such as the AR Binding Assay described in OCSPP Guideline 890.1150 and proposed by the EPA [39] as part of the Tier 1 of the Endocrine Disruptor Screening Program (EDSP). This test consists of a radioligand binding assay that identifies compounds able to compete for AR binding *in vitro*, and is not meant to measure the molecular intrinsic activity (α) and to classifying them as (partial) agonists or antagonists.

Tests aimed at the evaluation of the intrinsic activity (α) of putative EASs are mainly based on two end points: the measurement of cell proliferation or the use of an androgen-responsive reporter gene. The A-SCREEN assay [40] measures the proliferation of sensitive cells to screen for androgen activity. The MDA-kb-2 cell line has been developed by scientists at the EPA [41] through a stable transfection of AR and the insertion of an MMTV-driven luciferase reporter gene into the human mammary cancer MDA-MB-453 cell line. Both androgen and glucocorticoid agonists can activate the MMTV luciferase gene, and antagonists can be tested with respect to a fixed reference concentration of the agonist.

We carried out a validation of the molecular modelling and docking procedures we had devised by comparing experimental and computed dissociation constants (K_i_) for the human, rat and zebrafish endogenous hormones with respect to their ARs. A high *in silico* affinity between the endogenous ligands and their specific ARs is a *conditio sine qua non* for applying our selected approach to the investigated problem.

Experimental data available from scientific reports are associated with very high standard error of the mean. Computed binding free energies are evaluated from the analysis of specific ligand-receptor non-covalent interactions; their occurrence is associated with a score that can be interpolated on an experimental curve [42]. We have already reported that the use of empirical scoring functions for estimating dissociation constant values has accuracy in the range of one order of magnitude [43]. Furthermore, the specific ligand-induced activation mechanism of nuclear steroid receptors is based on a conformational transition, leading to the rearrangement of helix 12 [32], [33], which seemingly traps the ligand inside the binding site. Accordingly, the experimentally measured K_i_ have very low apparent values (corresponding to most favourable affinities). Conversely, the K_i_ values obtained through our *in silico* approach do not take into account such a displacement. This structural rearrangement is peculiar of nuclear steroid receptors, and should not be confused with the ligand-induced-fit process, which characterizes all the ligand-receptor interaction events. No induced-fit protocols [44] have been implemented in this investigation, since the ligand binding sites of the selected receptors were already well defined in the available crystallographic structures.

In this paper, our model was used to study the affinity of three fungicides (vinclozolin, iprodione and procymidone), as well as of their metabolites, with respect to the AR LBD of three different species (human, rat and zebrafish). These molecules were selected because of their classification in the same chemical group (dicarboximides) as well as of their classification as EASs. The mechanism behind the endocrine effects of both vinclozolin [10] and procymidone [16] is well-documented. They compete with the endogenous hormones for the binding to the AR, but they cannot activate it, because of their low intrinsic activity (α ≃ 0), and thus exert antiandrogenic effects. The toxic mechanism of iprodione has not been fully clarified yet and this compound is classified sometimes as antiandrogenic [16] sometimes as androgenic agent [14]. From our results, it is clear that all the tested fungicides and their metabolites can bind AR and compete with the endogenous hormones in all the tested species, exerting antiandrogenic effects.

In detail, in human and zebrafish, the tested compounds and metabolites can bind AR LBD with affinities comparable to the endogenous hormones: this suggests that there is a strong competition to occupy the binding site. On the contrary, the rat AR shows a lower affinity for the tested compounds, and - as a single assay – it is thus not a suitable molecular model to assess the toxicity of EASs and of their derivatives. However, identifying a toxic molecule in rat is an important alert signal, because this compound is likely to have an even stronger impact in humans.

## Conclusions

Our results shed a new light on the selection of the *in vitro* tests used for EAS hazard identification. Actually, rat seems to be less sensitive than human to the tested putative EASs. *In vitro* tests based on rat preparations could underestimate the sensitivity to these classes of molecules, differently from the human AR. On the other hand, zebrafish could be a more reliable model than rat, especially for environmental effects. For these reasons, the human AR LBD seems the most reliable target to be considered for estimating the EAS hazard in humans, whereas the zebrafish AR LBD should be considered, when environmental effects of EAS have to be investigated.

Our *in silico* approach emerges as a computational methodology for the evaluation of the AR affinity (prioritization of assessment) of a large number of molecules. During the design of new chemicals, the molecules that show highest affinities should in principle be disregarded and the main efforts should be focused on the molecules that show the lowest affinities. On the contrary, during a safety evaluation process, attention should be focused on the molecules that show the highest affinities.

**Figure 7:**
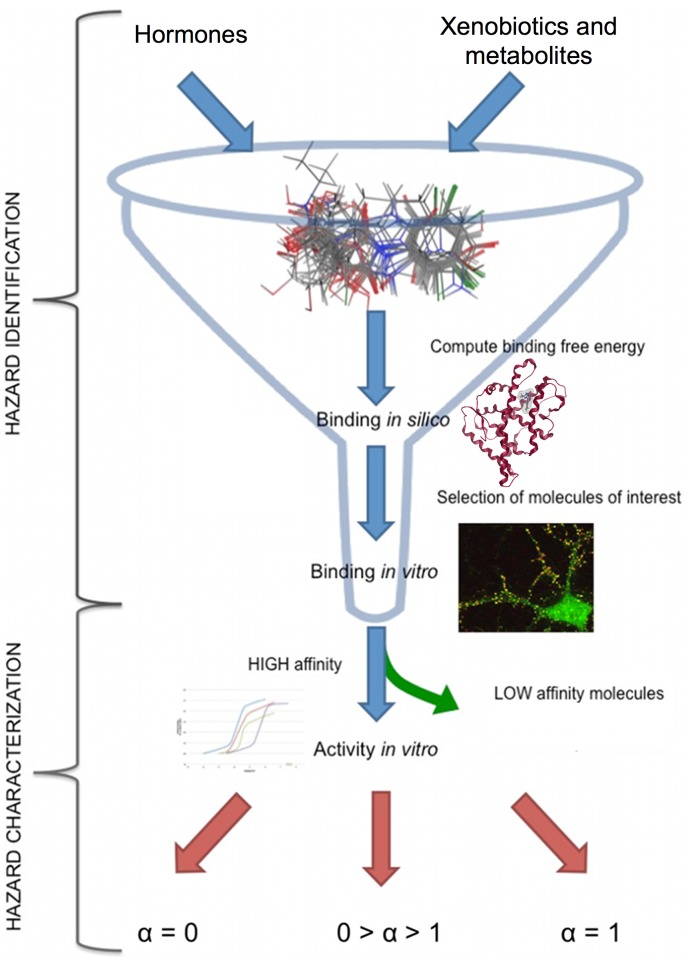
Hazard evaluation pipeline for putative androgen disruptors. Step 1: database production; step 2: *in silico* binding assay; step 3 *in vitro* binding assay for the selected dataset; step 4: *in vitro* activity assays only for the high affinity molecules (positive hits); and identification of agonist (α = 1), partial agonist (1<α<0) and antagonist (α = 0) activity.

While the *in silico* screening cannot be used as a stand-alone procedure, it can be successfully used as a first prioritizing step in a tier approach (Figure 7). The second mandatory check for the *in silico* positive hits should be an *in vitro* evaluation procedure, in which the affinity of the positive hits are measured through a reference cellular assay. From our results, the choice of the species to use in competitive binding assay should be carried out carefully, because it may lead to hazard over-under-estimation.

## Supporting Information

File S1 **The Supporting Information file contains:** **Figure S1.** Global alignment between primary structures of zebrafish and chimpanzee AR LBDs. **Figure S2.** Docking Score plot for the 1,000 non-interacting randomly selected compounds. **Figure S3.** Interaction network for DHT in the 3L3X crystallographic structure.
(DOCX)
