# Supplementary material for: A Computational Approach to Evaluate the Androgenic Affinity of Iprodione, Procymidone, Vinclozolin and Their Metabolites
Source: PLoS One. 2014 Aug 11;9(8):e104822. doi: 10.1371/journal.pone.0104822 (PMC4128724; doi:10.1371/journal.pone.0104822)
Supplement: File S1 — The Supporting Information file contains: Figure S1. Global alignment between primary structures of zebrafish and chimpanzee AR LBDs. Figure S2. Docking Score plot for the 1,000 non-interacting randomly selected compounds. Figure S3. Interaction network for DHT in the 3L3X crystallographic structure. (DOCX) [file pone.0104822.s001.docx]

***Supporting Information***

**A computational approach to evaluate the androgenic affinity of iprodione, procymidone, vinclozolin and their metabolites**

Corrado Lodovico Galli^§*^, Cristina Sensi^§^, Amos Fumagalli, Chiara Parravicini, Marina Marinovich, and Ivano Eberini

^§These authors equally contributed to this work^

**Figure S1**. Global alignment between primary structures of zebrafish and chimpanzee AR LBDs. Residues are colored by similarity according to the BLOSUM62 matrix.


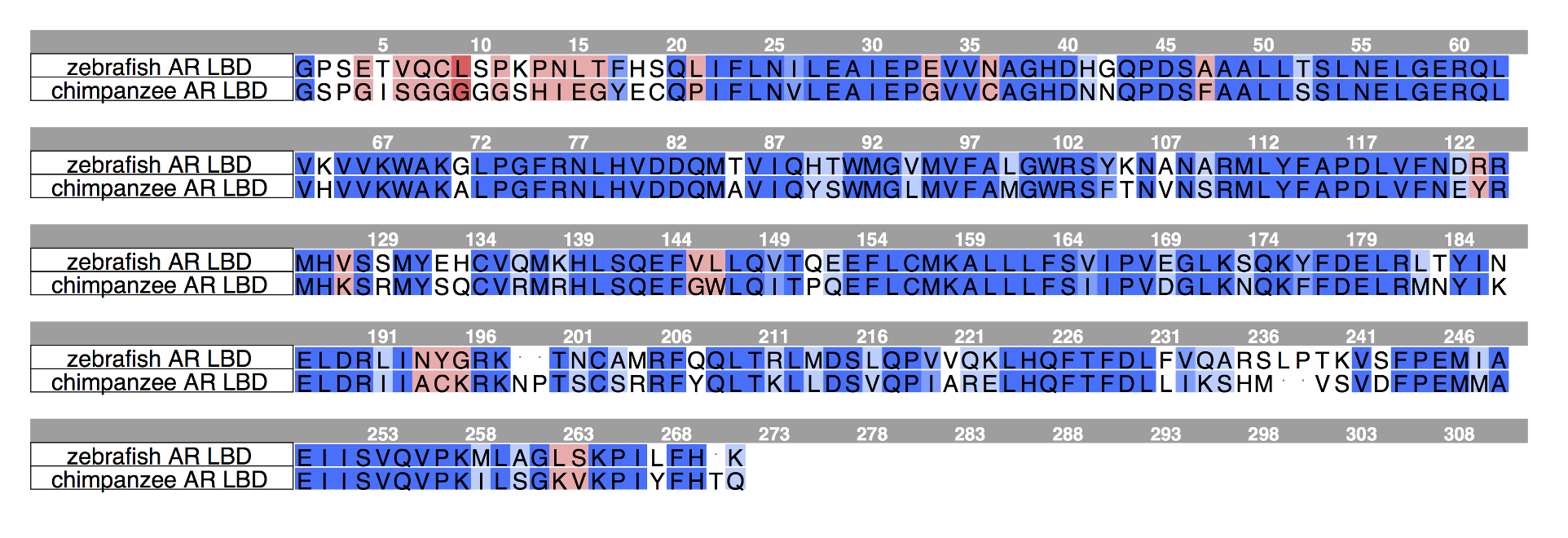


**Figure S2**. Docking Score plot for the 1,000 non-interacting randomly selected compounds.

**
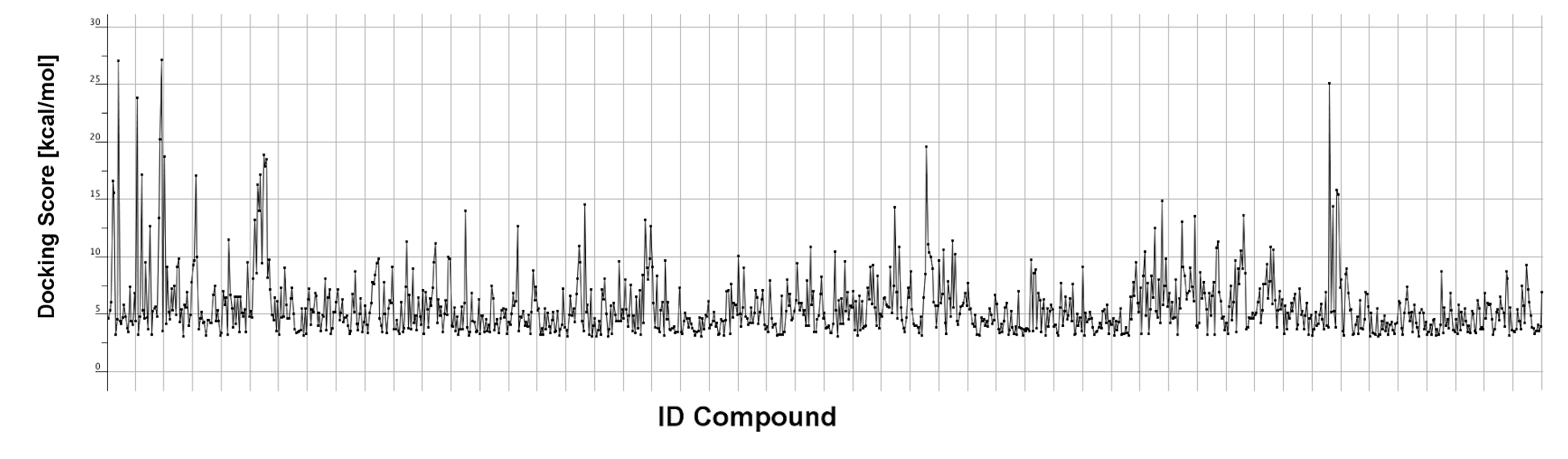
**

**Figure S3**. Interaction network for DHT in the 3L3X crystallographic structure; Met 895 is in the red box.

**
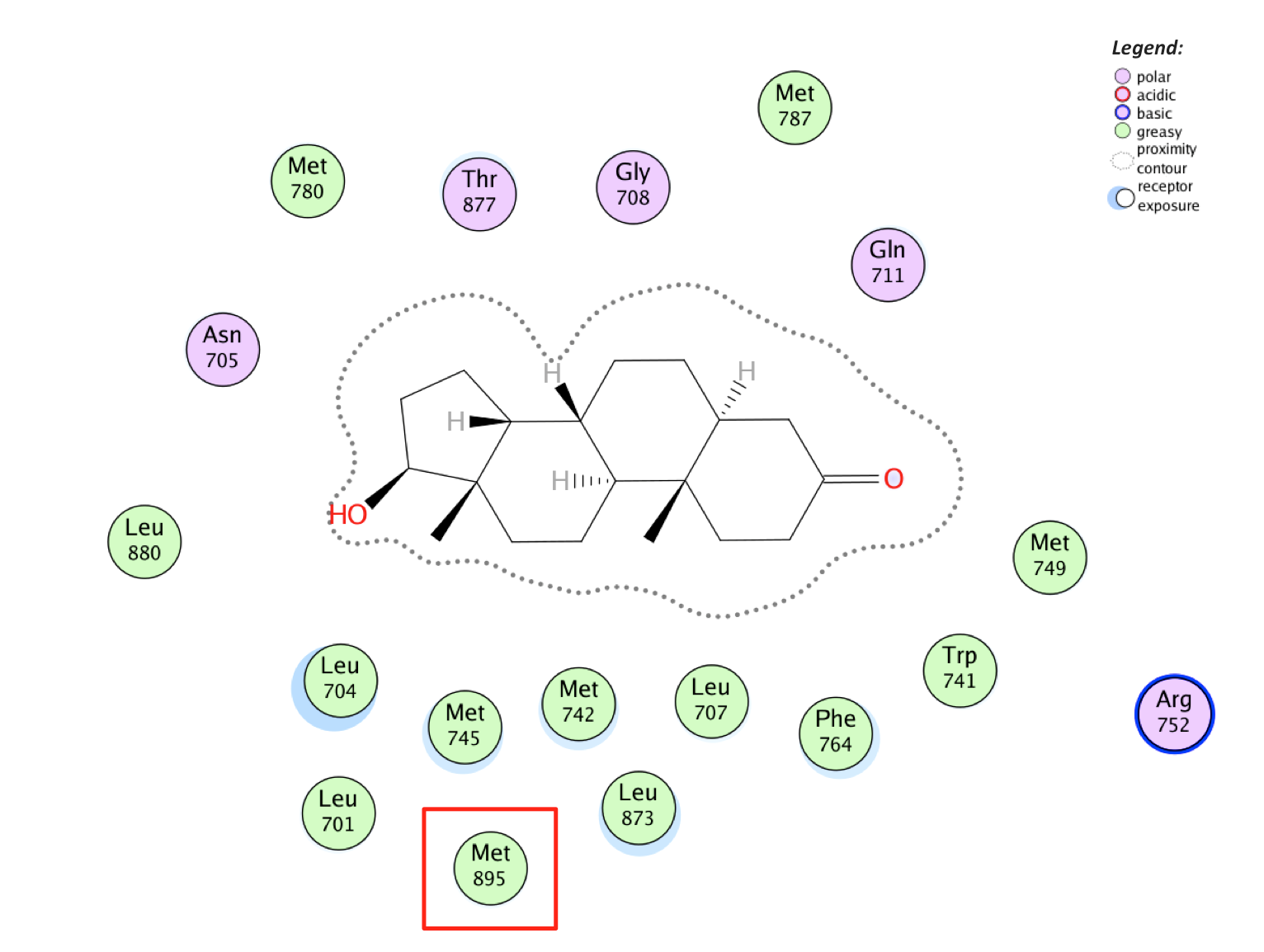
**
